# Response to: Reproducibility of the external surface position in left‐breast DIBH radiotherapy with spirometer‐based monitoring: methodological mistake

**DOI:** 10.1120/jacmp.v15i4.4954

**Published:** 2014-07-08

**Authors:** Aurora Fassi, Giovanni B. Ivaldi, Ilaria Meaglia, Patrizia Porcu, Paola Tabarelli de Fatis, Marco Liotta, Marco Riboldi, Guido Baroni

**Affiliations:** ^1^ Dipartimento di Elettronica Informazione e Bioingegneria Politecnico di Milano Milano Italy; ^2^ Department of Radiation Oncology Fondazione Salvatore Maugeri Pavia Italy; ^3^ Medical Physics Fondazione Salvatore Maugeri Pavia Italy; ^4^ Bioengineering Unit CNAO Foundation Pavia Italy

To the Editor:

We thank Siamak Sabour for his interest in our recently published manuscript.^(1)^ Our study was focused on the assessment of the reproducibility of the external surface position in repeated DIBHs controlled by a spirometric device. Patient surface data were obtained from an infrared (IR) optoelectronic localizer. Results were reported in terms of variability of the optically tracked surface 3D position within the same DIBH or among repeated DIBHs. The reliability of our measurements was verified by quantifying the accuracy of the tracking system in surface marker reconstruction, as reported in the paper.^(1)^ Since the measured variables do not depend on multiple observers or raters, the assessment of agreement proposed by the Author cannot be applied. Furthermore, the suggested ICC or weighted kappa coefficients are hardly applicable, since they are mainly used to assess the consistency of measurements made by different observers on the same quantity.^2^, ^3^ The second part of our study was focused on the assessment of dose distribution variations associated to the measured DIBH variability. The Author claims an inappropriate use of statistical tests in our study. We would like to reply that such criticism cannot be accepted, since no statistical tests were required to support our results and were reported in the paper. As a matter of fact, we verified the compliance of the obtained dosimetric variations with the planned dosimetric constraints, which have a relevant clinical importance.
